# Efficacy of Laryngeal Rehabilitation Therapy on Dysphagia after Anterior Cervical Surgery: Prospective, Randomized Control Trial

**DOI:** 10.3390/jcm11092470

**Published:** 2022-04-28

**Authors:** Jong-Hyun Ko, Kap-Soo Han, Sun-Jung Yoon

**Affiliations:** 1 Department of Orthopedic Surgery, Jeonbuk National University Medical School, Jeonju-si 54907, Korea; drhwata@gmail.com; 2 Research Institute of Clinical Medicine of Jeonbuk National University—Biomedical Research Institute of Jeonbuk National University Hospital, Jeonju-si 54907, Korea; hanks@jbnu.ac.kr

**Keywords:** ACDF, dysphagia, laryngeal rehabilitation therapy

## Abstract

Dysphagia is the most common complication of anterior cervical discectomy and fusion (ACDF). Several studies have reported dysphagia’s incidence, severity, and prognosis after ACDF; however, few have investigated the objective effects of dysphagia management. We aimed to elucidate the efficacy of laryngeal rehabilitation therapy for dysphagia following ACDF. This prospective randomized control trial included 20 patients who underwent more than two-level ACDF. Laryngeal rehabilitation therapy was performed on 10 patients for 7 days, whereas the remaining 10 comprised the control group. Pharyngeal transit time (PTT) by videofluoroscopic swallowing study (VFSS) was performed to evaluate the objective state of swallowing. We analyzed Bazaz scale and total variance of prevertebral soft tissue swelling (PSTS) from C2 to C7 on lateral cervical radiographs during hospitalization and at 4 and 8 weeks post-surgery. The PTT of the rehabilitation group was shorter than that of the control group at 7 days and 4 weeks post-surgery (*p*-value; POD 7D = 0.003, POD 4W = 0.042, POD 8W = 0.097). Perioperative laryngeal rehabilitation therapy effectively reduces postoperative dysphagia after ACDF.

## 1. Introduction

Anterior cervical discectomy and fusion (ACDF) is the most commonly used procedure in cervical spinal surgery. Although it is considered relatively safe, postoperative dysphagia is the most common complaint during the early stages after ACDF [1,2,3]. 

Many previous studies have reported the risk factors for postoperative dysphagia, such as the number of surgeries, operative time, age, sex, smoking, and plate profile [4,5,6,7]. Previous studies have introduced various strategies to reduce postoperative dysphagia, including local or systemic methylprednisolone therapy [8,9], endotracheal cuff pressure reduction [10], and application of tracheal traction exercise [11]. 

Various strategies to reduce dysphagia after ACDF have been introduced. These include modifying diet [12], applying voluntary control to swallowing [13], doing exercise to improve the range of oral or pharyngeal structural movement [14]. Recently, manual preoperative tracheal retraction was suggested, and the positive results were shown in reducing the occurrence of postoperative oropharyngeal dysphagia after the surgery [15].

In accordance with the previous efforts to reduce dysphagia after ACDF, this study aimed to examine the clinical efficacy of laryngeal rehabilitation therapy for dysphagia after ACDF and determine the relationship between subjective dysphagia and objective swallowing test via the videofluoroscopic swallowing study (VFSS).

## 2. Materials and Methods

### 2.1. Study Design 

#### 2.1.1. Trial Design

**Figure 1:**
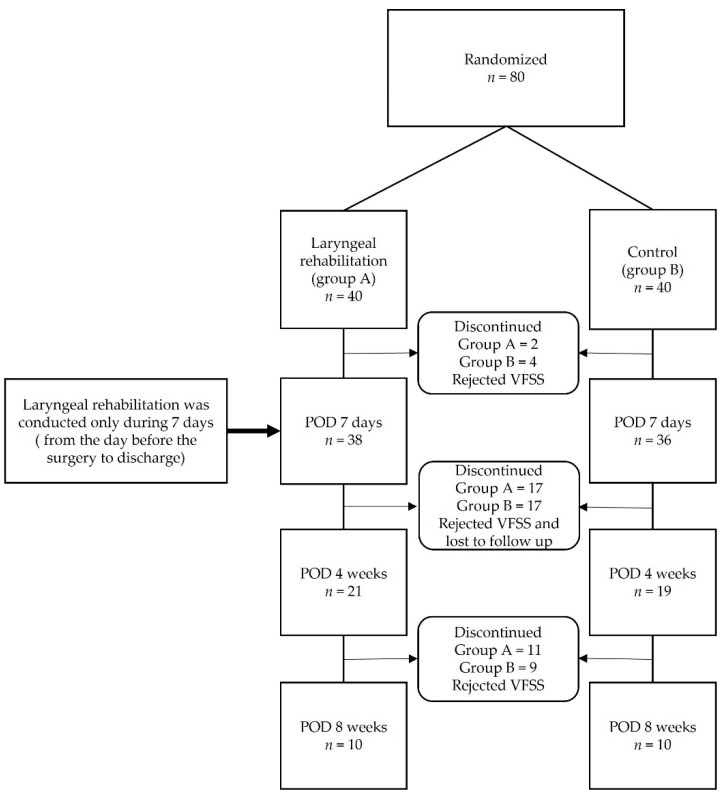
Study flow chart.

For this prospective randomized parallel control trial, we recruited patients with degenerative cervical spinal disease who underwent multilevel (more than 2 levels) ACDF between April 2015 to October 2016. All of surgery were conducted with left side standard Smith-Robinson approach. The study database included 80 patients with the degenerative cervical spinal disease who had undergone multilevel anterior cervical discectomy and fusion (more than two levels) with the same cervical plate (Maxima Anterior Cervical Plate System, U&I Corporation, Seoul, Korea) and fusion material (PEEK cage filled with DBM). In order to increase the statistical value such as parametric analysis, 40 students in each two groups, a total of 80 students, were enrolled by randomized selection. However, during the study, the patient’s compliance with the VFSS test was not high, so dropout occurred. Afterwards, during the outpatient follow-up period, VFSS was performed at 4 and 8 weeks of postoperative day (POD) to prove the effect of laryngeal rehabilitation, and a total of 20 subjects, 10 in each group, were finally enrolled (Figure 1). The laryngeal rehabilitation group (*n* = 10) received laryngeal manipulation by a physician, whereas the control group (*n* = 10) did not. We excluded patients with a history of anterior neck surgery, trauma, infection, tumor, neurological disorders associated with dysphagia (stroke or Parkinson’s disease), and poor compliance to traction maneuvers.

#### 2.1.2. Participants

Single tertiary medical center (JBNU) participated in this study. According to the trail protocol, inclusion criteria were male and female adults ≥18 years of age; diagnosed degenerative cervical spine disease, and receiving multilevel anterior cervical surgery (ACDF) by one surgeon with the same cervical and fusion. The perioperative treatment was conducted by JBNU anterior cervical spine surgery protocol (Table 1).

#### 2.1.3. Interventions

Included patients were randomized to either experimental group or control group according to laryngeal rehabilitation therapy.

Control group: Usual JBNU anterior cervical spine surgery protocol was conducted in this group without laryngeal rehabilitation therapy.

VFSS was used to objectively analyze swallowing function. Pharyngeal transit time (PTT; normal range; within 1.0 s) was checked to determine if there was a functional and structural delay in liquid swallowing. This study was performed with the day before surgery as the baseline, the seventh day after the surgery (POD 7, the day of discharge), and 4 and 8 weeks after the surgery.

#### 2.1.4. Randomization

##### Sequence Generation and Allocation Concealment

A computer-generated permuted block randomization sequence, stratified by hospital, was used for randomization purposes. A web-based platform allocated patients 1:1 to the study arms, randomly assigning a numerical code to each patient and the corresponding intervention. Researchers were blinded to the allocation sequence of the study interventions.

##### Implementation

Patients, selected after out-patient unit for degenerative cervical spine disease, were screened for inclusion and exclusion criteria, informed of the aims and purpose of the study, enrolled after signing the informed consent, and randomized to the experimental or control group. An out-patient unit coordinating nurse, who did not know the study design, generated the random allocation sequence.

### 2.2. Materials and Methods

#### 2.2.1. Laryngeal Rehabilitation Therapy

**Figure 2:**
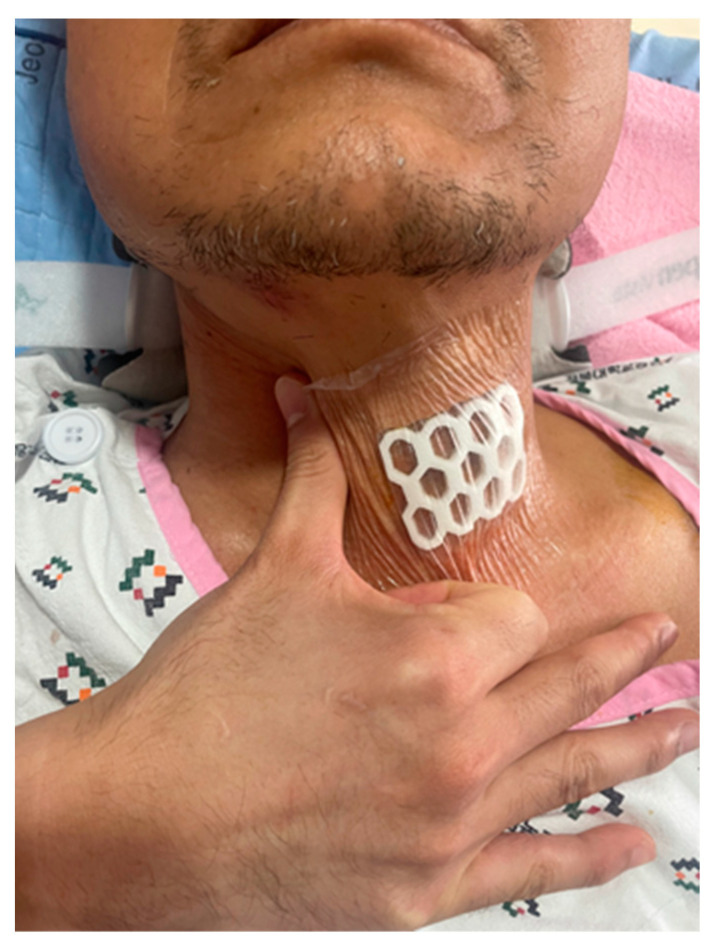
Laryngeal rehabilitation therapy: laryngeal manipulation. A physician pushes the patient’s laryngeal area including thyroid cartilage from the right to the left side. Voluntary swallowing is allowed by patients ten times at each cycle.

The rehabilitation therapy was performed by a physician at the rate of two cycles a day for seven days from the day before surgery to that before discharge (Video S1 in Supplementary Material). Laryngeal manipulation was performed by allowing the patient to voluntarily swallow ten times while pushing the patient’s laryngeal area, including thyroid cartilage from the right to the left side at each cycle (Figure 2). 

#### 2.2.2. VFSS (Videofluoroscopic Swallowing Study)

**Figure 3:**
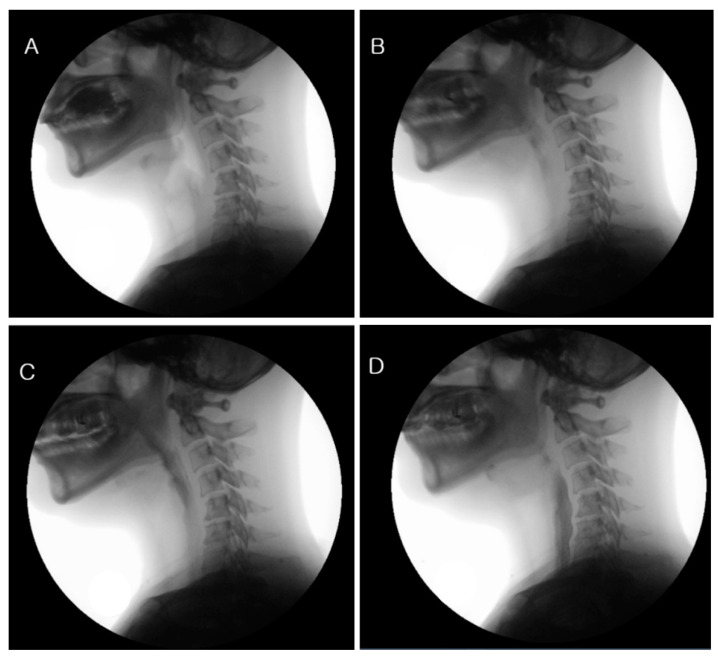
Pharyngeal transit time on VFSS. (**A**) An Oral phase. (**B**,**C**) Pharyngeal transit time is defined as the time it takes for the bolus to pass from the faucial arches over the base of the tongue and through the pyriform sinus in the esophagus. (**D**) Esophageal phase.

VFSS was used to analyze swallowing function objectively. Pharyngeal transit time (PTT; normal range; within 1.0 s) was checked to determine if there was a functional and structural delay in liquid swallowing (Figure 3). This study was performed with the day before surgery as the baseline, the seventh day after the surgery (POD 7, the day of discharge), and 4 and 8 weeks after the surgery. 

#### 2.2.3. Clinical and Radiologic Outcomes

**Figure 4:**
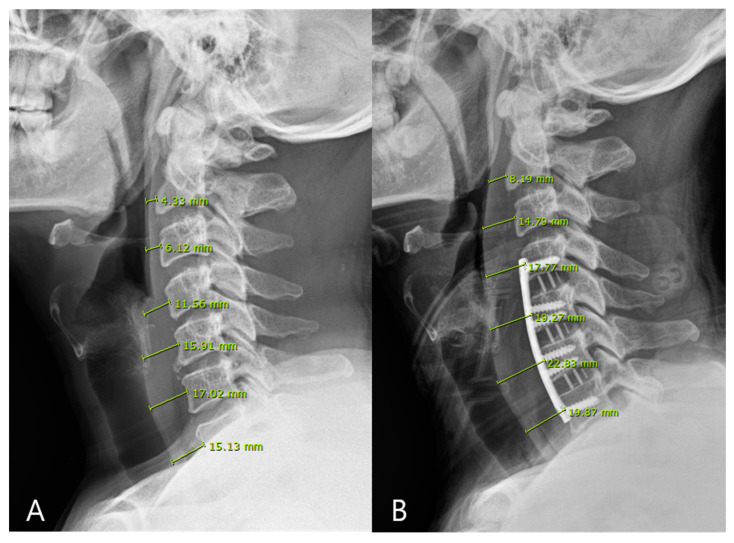
Prevertebral soft tissue swelling (PSTS) was calculated from the C-spine lateral radiographs. The sum of total variation in PSTS at each level from C2 to C7 was used to evaluate the relationship between PSTS and degree of dysphagia. (**A**) is preoperative PSTS at each level and (**B**) is postoperative PSTS at each level.

The degree of dysphagia was evaluated daily during the hospitalization period and at 4, 8, and 12 weeks after the surgery using the Bazaz scale, categorized as mild, moderate, or severe (Table 2). Prevertebral soft tissue swelling (PSTS) was measured using C-spine lateral radiographs, with the preoperative lateral view serving as the baseline. The PSTS was assessed daily using the lateral view until the date of discharge and that of follow-up to assess the total variance in soft tissue swelling at the C2–C7 levels (Figure 4). 

#### 2.2.4. Statistical Analysis

The two groups were compared to evaluate differences in the PTT on VFSS, the severity of dysphagia according to the Bazaz scale, the total variance of PSTS, age, sex, smoking history, and operative time. SPSS version 21.0 (IBM Corp., Armonk, NY, USA) was used. The Mann–Whitney U test and Fisher’s exact test were used. Statistical significance was defined as *p*-value < 0.05.

## 3. Results

The patient’s demographic characteristics, including age, sex, smoking history, operative time, and fusion level, did not significantly differ between the two groups (Table 3). 

### 3.1. VFSS

The PTT on VFSS was significantly shorter in the laryngeal rehabilitation group than in the control group at 7 days and 4 weeks after the surgery. However, there was no significant difference in PTT between the two groups 8 weeks after the surgery (Table 4). This result indicates that laryngeal rehabilitation therapy could shorten the passing time of the bolus and reduce the degree of dysphagia during the earlier postoperative period.

### 3.2. Clinical and Radiologic Outcomes

**Figure 5:**
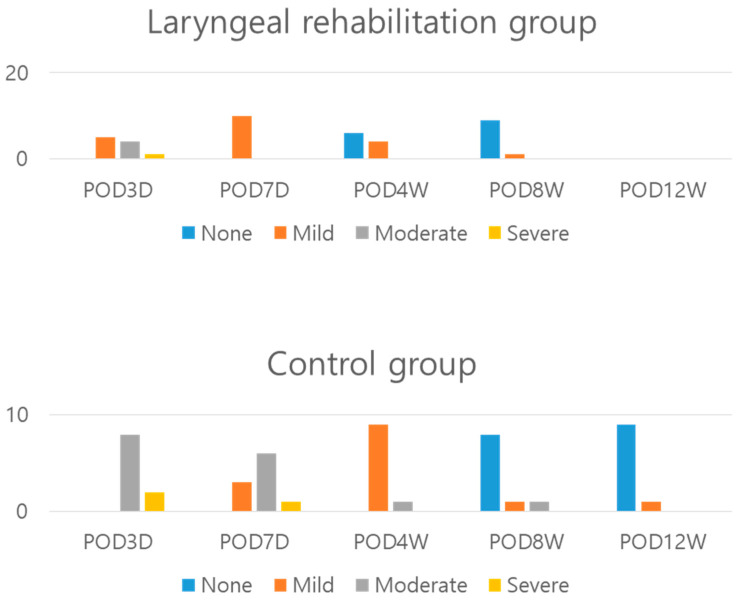
Dysphagia severity of patients in the laryngeal rehabilitation and control groups. POD, postoperative day; D, day; W, week.

The laryngeal rehabilitation group showed significantly greater improvement in dysphagia on the Bazaz dysphagia scale than the control group on the earlier days after surgery (Figure 5). However, there was no significant difference in the total variance of PSTS in the C-spine lateral radiographs between the two groups (Table 5). These results indicate that dysphagia may be more influenced by functional factors, such as swallowing muscles, than mechanical factors such as PSTS.

## 4. Discussion

Dysphagia is an uncomfortable side effect of patients and a common complaint among spine surgeons. In a prospective study of postoperative dysphagia after anterior cervical surgery, the incidence of dysphagia was 50.2% at 1 month and 12.5% at 12 months [16]. Despite this high incidence, several risk factors have been identified; however, the exact etiology of postoperative dysphagia remains uncertain [17]. 

Generally, the swallowing mechanism consists of oral, pharyngeal, and esophageal phases. Several muscles, soft tissues (such as tongue, palate, and pharyngeal and laryngeal muscles), and nerves are involved in swallowing [18,19]. In the normal swallowing process, sucking, chewing, and moving food or liquid into the throat occurs sequentially in the oral phase. At the pharyngeal phase, the swallowing reflex has occurred, and the food or liquid moves down the throat, and the airway is closed off to prevent regurgitation into the airway. During the esophageal phase, serial contraction of the esophagus is occurred to pass food or liquid into the stomach [20]. Dysphagia is a discomfort symptom indicative of an abnormality in the neural and/or muscular control of any phase of the swallowing mechanism or the mechanical obstruction [21,22]. 

Almost postoperative dysphagia after anterior cervical surgery can occur during the pharyngeal phase and the swallowing dysfunction can be divided into four categories: an inability or excessive delay in initiating pharyngeal swallowing, ingestate aspiration, nasopharyngeal regurgitation, and ingestate residue within the pharyngeal cavity after swallowing [23,24,25,26,27]. These categories are all related to the pharyngeal phase of swallowing mechanism. 

Dysphagia is a subjective symptom felt by the patient, so there is no objective sign, indicator, or measurement method. It is necessary to quantify the degree of dysphagia symptoms complained of by the patient and objective tests, but unfortunately, studies related to this are extremely rare. Therefore, the strength of this study is that the degree of swallowing function was objectively evaluated through the VFSS and relationship with dysphagia symptoms was attempted. The pharyngeal transit time of VFSS was based on, which was related to the pharyngeal phase in all four categories of postoperative dysphagia. 

Pharyngeal transit time (PTT) can be defined as the time it takes for the bolus to pass from the faucial arches over the base of the tongue and through the pyriform sinus into the esophagus is one of the most valuable parameters in VFSS for evaluating of dysphagia [23]. Delayed pharyngeal transit time is related to delayed elevation of hyoid bone and thyroid cartilage, which is highly associated with the retraction site during the ACDF procedure. In this study, we evaluated the PTT based on these reasons, and the result showed the relationship between subjective dysphagia and objective delayed PTT. 

The pathophysiology of dysphagia after ACDF have not well known yet, however, the causes of postoperative dysphagia thought to be multifactorial, which includes neuronal, muscular, and mucosal structures [5,13,28]. According to previous studies about the postoperative dysphagia, another possible factor affecting the occurrence and severity of postoperative dysphagia is PSTS [24]. However, PSTS may be the only mechanical factor in the swallowing mechanism. In our study, the laryngeal rehabilitation group had less severe dysphagia than the control group. However, the variance of PSTS was not significantly different between the two groups. Therefore, PSTS could be a possible risk factor but not an absolute risk factor in this study.

During anterior cervical surgery using the standard Smith–Robinson approach, the muscles, usually including the thyroid cartilage, related to swallowing are usually retracted to the right side by surgeons. According to our hypothesis, this procedure could cause temporary palsy of the swallowing muscles and its supplying nerves under the prerequisites; there is no definite neural injury related to swallowing, such as cranial nerves. Therefore, the laryngeal rehabilitation therapy in this study was designed as an opposite traction maneuver to the left side. In addition to left side passive traction, active swallowing was added to enhance the recovery of the temporary palsy. 

Our study has some limitations. Relatively, the sample size enrolled was smaller than some previous studies due to the nature of prospective clinical study. The difficulty with VFSS for swallowing function test was main reason to limit the sample size and brought the low compliance in the clinical test procedure. Many of enrolled patients were appealed their discomfort, whereas VFSS testing and dropped in the middle of the clinical test. In this study, Bazaz scale was used for dysphasia scoring due to its wide usage in previous study. However, this scoring method has some limitations such as clinician-administered, oversimplified, difficulties in swallowing solids and liquids and lack of validation [29]. Therefore, other scoring method such as MDSS (modified dysphagia scoring system) should be included to compensate Bazaz method in scoring dysphasia. Further study should be performed to ensure the conclusion with a larger sample size combining with other methods.

Laryngeal rehabilitation therapy can effectively reduce dysphagia in the early postoperative period. This can be supported as an improvement in the patient’s subjective symptoms based on the Bazaz scale and a more objective result based on the VFSS.

## 5. Conclusions

Although postoperative dysphagia is a non-fatal and self-limiting complication, it is the main complication that can reduce patient satisfaction at an earlier stage after surgery. In this study, it was shown that the efficacy of laryngeal rehabilitation therapy on the reduction in the severity of Dysphasia could be evaluated using the VFSS method.

## Figures and Tables

**Table 1:** JBNU anterior cervical spine surgery protocol.

Protocol	Description
Hospitalization Period	9 Days
Admission day	the day before the surgery
Discharge day	Postoperative day (POD) 7 days
High dose IV steroid administration 250 mg methylprednisolone	Every 6 h for 2 days (operation day and POD 1 day) to reduce the prevertebral soft tissue swelling for preventing airway complication or severe dysphagia
Closed suction drainage	Removal if the amount < 30 cc/day Usually 1~3 days after the surgery
Postoperative orthosis	Modified Philadelphia neck brace (Vista*^®^* Collar, Aspen, CO, USA) for POD 6 weeks
Follow-up protocol	Lateral X-ray: everyday check during hospitalization period
	AP and lateral X-ray: discharge day and regular follow-up day
	Regular follow-up: POD 4 weeks, POD 8 weeks, POD 12 weeks, POD 6 months, POD 12 months, and annual follow-up

**Table 2:** Bazaz scale for grading of dysphagia.

Severity	Liquid	Solid
None	None	None
Mild	None	Rare
Moderate	None/rare	Occasionally
Severe	None/rare	Frequent

**Table 3:** Patient demographics.

Variables	Rehabilitation Group (*n* = 10)	Control Group (*n* = 10)	*p*-Value
Age (mean ± SD), year	55.9 ± 9.6	54.6 ± 9.5	0.764 *
Sex (M:F)	6:4	4:6	0.660 ^†^
History of smoking	3 (30%)	3 (30 %)	1.000 ^†^
Operative time (mean ± SD), min	100.8 ± 20.1	98.6 ± 22	0.818 *
Levels of fusion	2 level = 2 3 level = 6 4 level = 2	2 level = 3 3 level = 4 4 level = 3	N/A

SD, standard deviation; M, male; F, female; N/A, not applicable. * Mann–Whitney U test. ^†^ Fisher’s exact test.

**Table 4:** Pharyngeal transit time on videofluoroscopic swallowing study.

	Rehabilitation Group (*n* = 10)	Control Group (*n* = 10)	*p*-Value
Preoperative (mean ± SD), s	0.90 ± 0.05	0.90 ± 0.07	0.849 ^†^
Postoperative, 7th day	1.34 ± 0.15	1.59 ± 0.18	0.004 *
4th week	1.08 ± 0.08	1.15 ± 0.06	0.028 *
8th week	0.91 ± 0.06	0.95 ± 0.04	0.112 *

SD, standard deviation; * Independent *T* test; ^†^ Mann–Whitney U test.

**Table 5:** Total variation and average of prevertebral soft tissue swelling on C-spine lateral radiographs.

	Rehabilitation Group (*n* = 10)	Control Group (*n* = 10)	*p*-Value *
POD 1D (mean ± SD), mm	88.6 ± 17.1	84.7 ± 18.2	0.591
POD 2D	92.2 ± 14.6	82.2 ± 19.2	0.200
POD 3D	90.4 ± 12.7	79.4 ± 21.8	0.184
POD 4D	88.7 ± 18.9	76.5 ± 14.6	0.128
POD 5D	83.6 ± 15.9	78.2 ± 14.3	0.431
POD 6D	80.8 ± 16.8	76.8 ± 17.0	0.600
POD 7D	74.3 ± 17.0	73.5 ± 19.0	0.920
POD 4W	54.8 ± 11.9	53.3 ± 12.6	0.780
POD 8W	50.4 ± 11.2	51.6 ± 10.8	0.800

SD, standard deviation; POD, postoperative day; D, day; W, week. * Mann–Whitney U test.

## Data Availability

Not applicable.

## Supplementary Materials

The following supporting information can be downloaded at: https://www.mdpi.com/article/10.3390/jcm11092470/s1, Video S1: Laryngeal rehab.
